# Can internal tobacco industry documents be useful for studying the UK alcohol industry?

**DOI:** 10.1186/s12889-018-5722-0

**Published:** 2018-06-28

**Authors:** Benjamin Hawkins, Jim McCambridge

**Affiliations:** Department of Health Sciences, University of York, Heslington, York, YO10 5DD UK

**Keywords:** Alcohol industry, Tobacco documents, Alcohol policy, Tobacco industry, UK

## Abstract

**Background:**

The release of internal documents now available in the Truth Tobacco Documents Library has offered important insights into the machinations of tobacco companies. These documents potentially offer additional insights into the nature of the alcohol industry, due to co-ownership and collaborative working across industries. This proof of concept study aims to build on the few examples of internal tobacco company documents being used to study alcohol industry activities, to identify the scope of information available on the UK alcohol industry.

**Methods:**

We identified the principal company names of the major national brewers, including predecessor company names, until the late 1990s, contemporaneous to the bulk of the tobacco documents. Using these names as initial search terms, we searched the Library to identify relevant material. Documents returned were then analysed for evidence of alcohol industry connections to the tobacco industry in the UK.

**Results:**

We found evidence of significant relationships between the two industries including previously unidentified data on co-ownership and cross industry shareholding; informal help-seeking between sectors; collaboration on issues of common interest; and cross industry ties via third party service providers, membership of common organisations and participation in shared events and platforms.

**Conclusions:**

These findings call for further research to analyse in greater depth the information identified here, and to explore alcohol industry activities and links with tobacco companies in other national contexts. This preliminary investigation suggests there is much valuable data available in the Truth Tobacco Documents Library that can serve to guide research on the alcohol industry.

## Background

Internal tobacco company documents offer researchers unprecedented insights into the ‘black box’ of tobacco industry strategies to oppose evidence based health policies [1]. For those working in the field of alcohol policy, the absence of similar repositories requires researchers to identify alternative data sources for examining industry activities. For example, documentary analyses and semi-structured interviews have been used to study alcohol industry actors within the United Kingdom (UK) [2, 3].

Given the sheer volume of information available in the tobacco documents library, and various connections identified to exist between the industries (e.g. co-ownership), scholars have sought to use these documents to gain insights into the alcohol industry practices [4–7]. However, this literature is limited, focusing principally on the co-ownership of Phillip Morris [PM] and the Miller Brewing Company [MBC]), and their activities in the US market. Bond et al. [5] found evidence of common regulatory concerns leading to collaborations between PM and MBC in developing political strategies. MBC feared increased taxation and additional regulation of their promotional activities, as well as negative public perceptions of their products, especially in relation to drink driving and ‘binge’ drinking [4]. To counter this, they promoted ideas of individual responsibility, which closely mirrored tobacco industry narratives [4]. As well as studying co-owned companies, Jiang and Ling [6] document how tobacco industry actors faced with a hostile policy environment have sought to build alliances with other industries, particularly alcohol, to oppose regulatory challenges in US tobacco policy that potentially also traverse sectors such as taxation and advertising restrictions. Dearlove et al. [7], meanwhile, catalogue tobacco industry attempts to recruit alcohol companies and the hospitality sector in the US to resist indoor smoking bans.

The UK alcohol industry experienced a process of significant consolidation in the second half of the twentieth century, especially up to 1990 [8–10]; a period for which there are substantial holdings of tobacco industry documents. This means company operating names changed and may be unknown to researchers unfamiliar with the UK context. In the same time period, tobacco companies undertook a process of diversification into (and divestment out of) different industries, including alcohol [11]. Given the 70 million pages of documents on the tobacco industry released, it appears likely that existing studies have not fully exhausted the resources available to alcohol industry researchers. Moreover, no previous studies have sought specifically to examine the UK context, or indeed other national contexts beyond the US.

The current paper seeks to address this evidence gap by identifying what information may exist within the Truth Tobacco Documents library in relation to the UK beer industry. A scoping study offers an opportunity to investigate proof of concept and consider the potential for further studies of this dataset. This research may be particularly informative about the nature of relationships between the two industries and, by extension, how far further research on the alcohol industry may benefit from what is already known about the tobacco industry.

## Methods

This study seeks to ascertain the scope of information available in the Truth Tobacco Documents Library on the UK beer industry, and thereby to consider the potential value of this data source for developing understanding of the alcohol industry. More specifically, we identified a priori a series of targets for data collection as being particularly informative about the nature of relationships between the two industries. We sought evidence on:

1. co-ownership between tobacco and alcohol companies;
2. shared facilities (i.e. office space) used between industries;
3. senior personnel moving between industries or working across industries simultaneously (e.g. individuals with concurrent directorships in both industries);
4. overlapping policy/ business concerns and collaboration between industries in developing strategic responses;
5. assistance-seeking by companies in one industry from actors in the other industry;
6. shared service providers (e.g. supply chain, accounting, legal, public relations and other professional service providers) between companies and across industries;
7. membership of third party bodies (e.g. campaign groups and trade associations);
8. attendance at meetings, conferences, shared forums or other events.

The methods for conducting tobacco documents research are well established (see [12]). We began with an initial keyword search of the Truth Tobacco Documents Library using the ‘Guided Search’ facility to search for brewing company names using the ‘Organization’ search tab. As our focus was the UK, a predetermined list of company names was developed from historical studies of the UK beer industry. The initial search terms were company names found in overviews of the UK beer industry contained in Gutzke [9] and Gourvish and Wilson [8]. All available libraries and collections present in the Truth Tobacco Library database were searched. No time parameters were imposed on the searches. Multiple variations of names were used (e.g. ‘Scottish and Newcastle’ and ‘Scottish & Newcastle’). Initial searches generated a large volume of documents, which were reviewed for relevance in an exploratory stage. In keeping with established methodologies [12], where larger numbers of documents were found, the returns were sorted by relevance and the first 60–100 documents reviewed, followed by a random selection of subsequent documents. Tobacco documents work requires a trade-off between the exhaustiveness and breadth of analysis, given the sheer volumes of material available in the database.

A decision was taken to focus our analysis in this preliminary investigation on the ‘Big-Six’ brewers which were dominant in the UK by the 1980s – Allied-Lyons, Courage, Scottish and Newcastle (S&N), Bass-Charrington, Grand Metropolitan (Grand Met), and Whitbread – and the predecessor and constituent companies from which these were formed (e.g. Newcastle Breweries, Watney’s). We also examined Guinness, which merged with Grand Met in 1997 to form Diageo, the world’s largest alcohol company at the time. In 1994, Allied-Lyons merged with Pedro Domecq S.A. for form Allied-Domecq, which was subsequently acquired by Pernod-Ricard in 2005. These mergers signal the breakdown of the previously clear separation between brewers and spirits producers, and the emergence of multi-category alcohol corporations, which now dominate the global alcohol market.

Searches were run iteratively to refine the search terms and excluding confounding terms. The optimum search terms for each company were decided, taking into account the volume of hits and to identify ‘saturation points’ (after which useful material is unlikely to be present) [12]. The final set of search terms used and the number of documents returned for each company are shown in Table 1 below.

**Table 1:** Second Stage Search Terms and Documents Returned

Company	Search Terms [Tab]	Documents Returned
Allied Breweries	“Allied Breweries” [Organization]	63
Allied Lyons	“Allied Lyons” [Organization]	221
Allied-Domecq	“Allied-Domecq” [Organization]	149
Courage	“Courage” [Organization]	231
Scottish & Newcastle	"Scottish & Newcastle" [Organization]	167
Newcastle Breweries	"Newcastle Breweries" [Organization]	31
Bass- Charrington	“Bass” [Organization] AND “Bass” [Everywhere] AND (“beer” OR “brewery” OR “ale”) [Everywhere] ~10^a^ NOT (“Roger” OR “fish” OR “bassmaster”) [Everywhere]	206
Charrington	“Charrington” [Organization]	24
Watney and Co.	“Watney” [Organization]	68
Whitbread	“Whitbread” [Everywhere] AND (“beer” OR “brewery”)~10^a^ [Everywhere]	98
Guinness	“Guinness” [Organization] AND (beer OR brewery) [Everywhere]	223
Grant Metropolitan	“Grand Met” OR “Grand Metropolitan” [Organization] AND ("UK" OR "GB" OR "Ire") [Everywhere] AND ("alcohol" OR "drink") [Everywhere]	337

^a^This symbol is used in the Truth database to indicate presence of search terms within 10 words of each other

All documents returned were then accessed and read. All types of document (e.g. letters, memoranda, annual reports) which related to policy relevant activities of the UK alcohol industry were included in the third and final stage of study. Where documents focussed on countries other than the UK (principally the US), these were included as potentially offering wider insights into the modus operandi of these companies, which are increasingly global in character [13]. Duplicate documents and ‘restricted’ documents (which are listed in search returns but cannot be opened and read as the contents were deemed privileged) were included in the number of search returns but were not examined. In total 169 documents were included in the data analysis. The pdfs of included documents were downloaded, saved and read in detail. Information relating to each company was extracted and tabulated. Data analysis involved summarising the nature of the information available in relation to each of our a priori targets for data collection. As these documents are all in the public domain, ethical approval for the study was not required.

## Results

The study findings are presented in each of the target data collection areas identified a priori for study, with the exception of shared facilities where we did not identify any relevant data. We found information on shareholding across industries, as a well as more formal and extensive co-ownership. As the former implies a different type of relationship to the latter, we present these two separately. Where necessary for clarification, details of third parties (i.e. companies, associations, membership organisations) are given in parenthesis. A limited amount of material contained within the analysed documents on the activities of the alcohol industry (principally to do with marketing strategies) is unrelated to connections with the tobacco industry, and is not presented here.

### Co-ownership between the tobacco and alcohol industries

There are a number of examples of co-ownership between alcohol and tobacco companies, some of which have been the subject of previous analyses [4–6]. Here we focus on two examples, which, as far as we are aware, have not been discussed in the research literature. Between 1970 and1986 the major national brewer Courage was an entirely owned part of the Imperial Group based around the tobacco company of the same name. Documents mainly relate to the internal structure of the company and the movement of executives between its alcohol and tobacco divisions, leading to a cross fertilisation of management practices, corporate culture and strategy. As the Imperial Group review from November 1972 notes the integration of the brewing interests within the overall structure of the company was facilitated by the appointment of Courage Executives to the Imperial Group Board [14]. These data extend previous findings about the MBC and PM [4, 5], indicting a higher level of integration of alcohol and tobacco strategic operations in this instance of co-ownership.

The second example of co-ownership between industries was Grand Met’s 1980 acquisition of US tobacco company Liggett and Mayers (L&M). Grand Met sold L&M in 1986, a year before it merged with Guinness to form Diageo. It is not clear whether these events were related. While Grand Met saw few long term prospects for their tobacco investments, in the short term it provided “a source of cash that will be reinvested in their other lines of business- hotels, food, liquor, etc” [15]. The other motivation for the takeover was to assume control over L&M’s wine and spirts businesses in the US (*Carillon* and *Paddington*). Grant Met’s 1982 annual report described the merger as providing “a significant presence in the US domestic market for branded consumer products” and consolidating its wines and spirits business by adding US distribution networks [16]. Entry into the tobacco sector with the purchase of L&M was an early move in the process of consolidation and globalization of the alcohol corporations, which now characterises the sector [13]. Its 1981 annual report describes this as giving “fresh impetus to the international development” of the group’s activities [17]. The L&M example shows that patterns of co-ownership may be complex and secure a range of advantages in the development of corporate strategy.

### Shareholdings and investments across industries

There were multiple examples of shareholding and investments across industries. In some instances, this established co-operative relationships between companies, for example between British American Tobacco (BAT) and S&N. In a letter dated 19 October 1988, BAT Chief Executive Pat Sheehy was asked by S&N Chairman Sir David Nickson to discourage his organisation’s pension fund from selling shares held in S&N to ward off a hostile takeover. Sheehy and his Board were also asked to lobby the UK government on behalf of S&N, given the ‘wider political issues’ that arise from the bid and ‘the consequences that follow when an important part of British industry falls under the control of a larger group with a distant head office’ [18]. These exchanges indicate close and informal relationships at the time between senior executives in the alcohol and tobacco sectors. However, it is unclear, in the absence of additional data, how far BAT’s shareholdings influenced the working relationships between companies. The direct connections between shareholding and political strategies identified here are noteworthy.

### Senior personnel working in both industries

The documents examined contained evidence of senior executives moving between industries or working across industries within companies where there was co-ownership, though this is not limited to co-ownership. Board memberships across the industries are identified which are independent of co-owned companies. These are summarised in Table 2 below, which details individuals holding directorships across industries. Dates given relate to the earliest available date of co-directorships, which are given in the relevant documents, and examples are ordered chronologically.

**Table 2:** Cross-industry directorships

Name (Date)	Alcohol industry connections	Tobacco industry connections
M. A. Anson (1974)	Director, Courage Ltd.	Director of Imperial Group Ltd. and Assistant Managing Director of Imperial Tobacco Ltd.
Mr. R.O. Steel (1974)	Director of Courage Ltd. and Chairman of Courage (Eastern) Ltd. and Courage (Central) Ltd	Director of Imperial Tobacco Ltd. [Additional co-directorships in Imperial Group are evident]
Stuart D. Watson (1983)	Former Chairman of the Board of Heublein was newly elected. Board Member Allied-Lyons PLC	Board Member, RJ Reynolds
Sir Alick Rankin (1993)	Chairman and former Chief Executive, Scottish and Newcastle Breweries Plc Director, The Brewers’ Society	Non-Executive Directors, BAT Member of the Nominations and Compensation Committees
Mr M. Luce (1994)	Brand Marketing Director, Courage Ltd.	Consumer Marketing Manager, BAT Latin America (Colombia)
Gerald Thorley (undated)	Director, Allied Breweries Plc	Director, BAT

### Overlapping policy and/or commercial concerns

The main issue of common concern across industries was the issue of environmental tobacco smoke and introduction of clean air legislation in the UK and the US. In the UK, S&N, which remained a major operator of licenced premises until around 1990, were also affected by the issue of smoke-free policies. S&N found themselves the subject of lawsuits from bar workers who claimed their health had been adversely affected by working in smoke filled environments and that the company had abdicated its duty of care to its employees [19]. In the US, the AtmospherePlus programme funded and driven by Phillip Morris sought to engage alcohol producers, the hospitality industry and relevant trade associations across these sectors to oppose smoke free bars [20]. Bar and restaurant operators, many of which were part of larger companies including global alcohol producers, were presented as innocent parties whose interests would be adversely affected through misguided and excessive forms of regulation.

In addition to common policy issues, there is evidence of emerging corporate social responsibility (CSR) activities in the alcohol sector, drawing on the experience of such initiatives in the field of tobacco. For example, the 1981 Grand Met annual report [17] states that:

a new company, Grand Metropolitan Community Services Ltd, has been formed with the sole objective of mitigating the problem of unemployment in the UK-particularly amongst the young." Staff have been seconded to the venture and charged with the task of identifying projects, which would create employment, and then supervising their implementation. Good progress has already been made, in co-operation with various government departments and other sponsors.

Similarly, the 1982 Annual Report [16] cites attempts by the company to contribute to policy debates and to highlight the contribution of the industry to society, which policy makers and the public are not sufficiently aware:

In most countries, IDV [International Distillers and Vintners] and other drinks companies are not only making positive contributions to research and other work on social aspects of alcohol, but are also making, through heavy excise taxes, large contributions to national exchequers. Few governments recognise this fully, and we are hoping that the recent concession in the timing of duty payments will mark the start of a more constructive dialogue with Government in the UK.

These objectives foreshadow the later, more developed political strategies identified as being employed by the alcohol industry in more recent policy debates [21, 22]. Tobacco documents are thus an important source in contextualising current analyses of alcohol industry political activity and demonstrating both the origins and longevity of industry strategies, which mirror those of the tobacco industry.

### Assistance seeking between industries

In addition to the material presented on assistance seeking in the section on shareholding, relevant material in this category related principally to solicitations between industries for sponsorship of supported projects or to development of, and expertise sharing on, marketing and corporate social responsibility (CSR) activities. For example, Allied executives sought funding from BAT for sponsorship of the Glyndebourne festival [23], whilst BAT asked Whitbread to raise funds and secure the holdings of the Royal Commonwealth library [24]. Similarly, BAT set out proposals to for Guinness to sponsor the world powerboat series, which the tobacco company were also sponsoring [25]. We thus identified no further clear examples of political collaborations in addition to those previously reported.

### Use of common service providers

There are many examples of tobacco and alcohol companies using the same professional services providers, particularly in the marketing sector. Table 3 details contacts between third party service providers and the alcohol and tobacco industries along with details of the type of organisations mentioned and the earliest available date of contacts with the alcohol/tobacco industry in the documents examined. Examples are presented in chronological order. The level of detail available on the services undertaken by the companies cited above for the alcohol and tobacco industries varies between documents. It is not possible to understand the precise nature of the relationship of service providers with each industry, and the connections this may have fostered across industries, without further study. Where specific details of these connections with alcohol and tobacco industries are available, these are discussed below.

**Table 3:** Common Service providers

Service Provider (Description; Date)	Alcohol industry	Tobacco industry
Law Society Commerce & Industry Group (1977)	Group member companies have multiple alcohol industry clients	Group member companies have multiple tobacco industry clients
Nitrosamine processing laboratories (1979)	Allied	BAT
Keenan McLaughlin Inc. (Advertising Agency; 1981)	Bass	Cigarette Warning Project, unspecified tobacco industry clients
Research Disclosure Magazine (1982)	Watney	BAT
Bank of New York (1989)	Allied, Guinness	BAT
Shook Hardy & Bacon (Law Firm; 1990, 1998)	Guinness	American Brands, Brown and Williamson, Phillip Morris, RJ Reynolds
First Magazine (International Affairs Journal; 1993)	Multiple alcohol industry actors	Tobacco Advisory Council
International Advertising Association (IAA; 1993)	The Scotch Whisky Association (Edinburgh), Centre for Information on Beverage Alcohol (London), the Federation Internationale des \Vine at Spirtueux (Paris), Distilled Spirits Council of the US (DISCUS, US), Anheuser-Busch, Seagram, Heineken, Allied Lyons, Bacardi International, Guinness, Heublein, Hiram Walker-Allied Vintners, IDV, Whitbread.	Phillip Morris and other unnamed companies
Raeburn Keslake, (Corporate Affairs Training Provider; 1995)	Whitbread, Grand Met, United Distillers, Ushers Brewery	BAT, Kraft (Philip Morris)
Interbrand (Global Branding Consultancy; 1995)	Anheuser-Busch, Bass, Bacardi, Heineken, Guinness, Grand Met, Miller, Seagram	Japan Tobacco, Philip Morris, Brown and Williamson, RJ Reynolds
Mindy Goldberg Associates (Market Research & Analysis; 1998)	Bass, Guinness, Seagram	RJ Reynolds, RJR-Nabisco, Kraft (Philip Morris)
Field Marketing Inc. (1999)	Allied Domecq, Guinness, Coors, Brown Forman, Bacardi-Martini	Philip Morris, USA
Marketing Connections (Consultancy; 2000)	Guinness Import Co. (Bass, Pilsner Urquell) & Jose Cuervo	Newport, Camel/ Salem, Benson & Hedges, Marlboro, Virginia Slims (Philip Morris)
Cambridge Group (Business Strategy Consulting; 2000)	Guinness	Lorillard
GIRA (research consultancy; undated)	Bass-Charrington, Hueblein, Kronenbourg, Labatt, Scottish & Newcastle	BAT, Imperial, Reemstma

Most details are available in the cases of First Magazine, the International Advertising Association (IAA) and the law firm Shook Hardy and Bacon. *First* had previously published reports on the alcohol industry and alcohol-related issues sponsored and supported by various industry actors. It approached the Tobacco Advisory Council about funding similar reports on issues relevant to the tobacco industry. Documents examined indicate that the IAA had undertaken previous, successful, advocacy campaigns and engagement activities on behalf of various alcohol industry actors. It details also advocacy on key tobacco industry issues such as advertising and sponsorship restrictions for various tobacco companies including Philip Morris. Shook Hardy and Bacon engaged in dialogue regarding the recruitment of ‘independent’ experts to promote favourable policy positions related to alcohol consumption in line with previous tobacco industry activities. Collaborations involving third party service providers in politically sensitive areas represent another potential mechanisms by which important policy learning may be transferred across industries.

### Membership of 3^rd^party bodies

Table 4 sets out details of organisations featured in the analysed documents which had both alcohol and tobacco industry members, listing the companies from each sector, and which were members of each. The earliest available date of membership is given and used to order examples chronologically. Where clarification of the type of body is needed this is given along with the date of documents detailing cross-industry contacts. These include highly policy relevant bodies.

**Table 4:** Cross-industry memberships of 3^rd^party bodies

Body/ Association (Date)	Alcohol industry	Tobacco industry
Biochemical Society (1964)	Bass, Charrington, Distillers, Watney-Mann, Whitbread;	BAT, Imperial
British Industrial Biological Research Association (BIBRA; 1968, 1969, 1970,1981, 1982 1994–5)	Brewers’ & Licenced Retailers Association, multiple small local and large transnational brewers (including Courage, Kirin Brewery)	BAT (UK & Export), Imperial, Rothmans
Industry Council for Research on Packaging and the Environment (INCPEN; 1978)	Allied, Bass-Charrington, Distillers, Guinness, Scottish & Newcastle	Imperial
Tobacco Duty Free Group (1988, 1989)	United Distillers, Allied, Hiram Walker, Seagram	Imperial, BAT, Gallaher, Rothmans, Philip Morris
Chicago Advertising Club/ Federation (1991)	Guinness, Miller Brewing Corporation, Distilled Spirits	Multiple tobacco industry members including Tobacco Institute &Philip Morris
Public Affairs Council (Cross Sector Body; 1992)	Grand Met	Philip Morris
UK Federation for Culture (1993)	Allied, Bass, Scottish & Newcastle, Whitbread	BAT
World Federations of Advertisers (1993)	Guinness, Allied (Allied-Domecq), Grand Met, Seagram, Diageo, Heineken	PM, BAT, Rothmans
Proshare (Share Investment Club; 1994)	Guinness, Grand Met, Scottish & Newcastle	BAT
Associates for Research into the Science of Enjoyment (ARISE; 1994)	Guinness	BAT, RJR, PM, Rothmans
Biological Council (Undated)	Allied, Bass, Watney, Whitbread	BAT

### Attendance at same events

There were a range of different types of formal events attended by both alcohol and tobacco company representatives or endorsed and sponsored by these companies (see Table 5 below). These potentially offer forums for engagement and cross-fertilisation of strategies and ideas across sectors. Meetings and events organised by membership bodies detailed in Table 4 above and so are not included here. The extensive nature of the contacts of BAT is apparent in these data.

**Table 5:** Attendance by tobacco and alcohol industry actors at the same formal events

Meeting/ Event (Date)	Alcohol industry	Tobacco industry
Informal Discussion meeting for Chairmen & Directors of Large Organisations held at BAT offices (1966)	Courage, Barclay Simmonds	BAT, Gallaher
Practical Skills of Managing People (1981)	Allied, Bass, Charrington, S&N	BAT
Practical Skills of Managing Event (1981)	Allied, Bass-Charrington, Scottish & Newcastle	BAT
Fermenters Symposium (1982)	Allied, Watney, Grand Met	BAT
CBI Conference, Seminar: Employee Involvement (1983)	Allied, Bass, Brewers’ Society, Grand Met, Lyons Tetley, Truman, Whitbread	BAT
Institute of Chemical Engineers (ICHEME) Meeting (1985)	Guinness, Watney, Nabisco	BAT
Minerva Public Relations Group UK Meeting (1988)	The Alcohol Duty Group	The Tobacco Duty Free Group
Statistics for Industry Ltd., Statistical Workshops (1991)	Allied, Bass, Guinness	Imperial, Rothmans
Advertising Association- Performance Monitoring Seminar (1994)	Carlsberg-Tetley, Allied-Lyons, Hiram Walker Group, United Distillers	BAT
Monitoring Advertising Performance Event (1998)	Guinness, Scottish Courage, IDV	BAT

## Discussion

This study considers the possible value of the internal tobacco company documents during an important period in the evolution of the emerging global alcohol industry. The documents examined show evidence of significant inter-connections between the alcohol and tobacco industries. Most obviously, this occurred in the form of various instances of co-ownership between sectors, which led in turn to integration of company functions and cross fertilizations of corporate strategy, also aided by common directorships across industries. Corporate cultures, practices and strategies developed within tobacco companies, or through close association with tobacco divisions within alcohol companies, may have diffused widely through the alcohol industry (and vice versa) as suggested by previous studies of the alcohol industry [3, 13, 26, 27]. The specific substance of the interconnections between industries both in the UK and beyond warrants further attention in studies of alcohol industry strategies to advance interests and shape policy.

In addition to the key findings on co-ownership, there are three further observations that can be made about these inter-connections on the basis of this study. Firstly, there are strong informal contacts between industries that serve as the foundation for an array of reciprocal help-seeking activities. These function in similar ways to the cultivation of long-term inter-personal relationships by the alcohol industry with policy actors previously identified [3, 21]. Moreover, these forms of collaboration appear likely in areas of common commercial interest. This finding mirrors those of previous studies [6, 7]. Secondly, the relationships between the alcohol and tobacco sectors needs to be considered within the broader context of cross sector corporate collaboration [28–30], including membership of third party bodies, attendance at the same meetings and events, and the use of common professional service providers. Thirdly, BAT appears to be particularly prominent and important to study carefully in future investigations of cross industry connections.

This study adds to the existing proof of concept. The Truth Tobacco Documents Library represents a valuable and under-utilised data source for scholars seeking to understand the historical evolution of the alcohol industry, and so to locate the current activities and strategies employed by alcohol companies in their historical context. The tobacco documents offer a resource for examining individual alcohol industry actors and organisations with no previously established connections with the tobacco industry.

The insights presented and discussed here may be further developed through triangulation with other data sets. Previous experience with tobacco documents research underlines the importance of ‘snowball’ techniques in the identification of additional search terms to identify further relevant documents. Further searches may also focus on product brands, for example, rather than company names. Similarly, searching consecutive Bates Numbers for relevant documents may uncover further useful texts.

This study focussed largely on the UK beer sector. It is likely, therefore, that additional information on UK spirits companies is contained within the Truth library. Similar studies on alcohol companies in other countries should be fruitful to undertake, though we make this suggestion without implying that further research should take place only at the national level. This study scopes available information rather than analysing it in depth. Our study was also circumscribed in terms of the data we sought, focussing on connections between tobacco and alcohol companies. As with all studies of the tobacco documents, the insights derived here are of a largely historical nature with the most recent documents identified dating from the early 2000s. This tobacco company research resource offers, nonetheless, the capacity to develop new insights both of a historical nature and with clear contemporary relevance into the alcohol industry.

## Conclusion

There is highly relevant material in existence in the Truth Tobacco Documents Library on the alcohol industry, which may provide a valuable data source for researchers interested in better understanding the alcohol industry and its relationships to the tobacco industry. Much of the data we have examined pertains to the period leading up to the consolidation of the global alcohol industry, and offers insights into developments in this key period, which have important health implications for understanding the political significance of the industry today. This preliminary study has identified data that can inform decision-making about future studies. It remains to be seen just how far further research on the alcohol industry may benefit from what is already known about the tobacco industry.
